# Seminal plasma proteins and metabolites: effects on sperm function and potential as fertility markers

**DOI:** 10.21451/1984-3143-AR2018-0029

**Published:** 2018-08-03

**Authors:** Arlindo Alencar Moura, Erdogan Memili, Antônia Moemia Rodrigues Portela, Arabela Guedes Viana, Ana Luiza Cazaux Velho, Maria Júlia Barbosa Bezerra, Fábio Róger Vasconselos

**Affiliations:** 1 Department of Animal Science, Federal University of Ceará, Fortaleza, CE, Brazil; 2 Department of Dairy and Animal Sciences, Mississippi State University, MS, USA

**Keywords:** fertility, metabolites, proteins, seminal plasma, sperm.

## Abstract

Molecular components of sperm and in the media surrounding them influence male fertility. In this regard, seminal plasma proteins and metabolites modulate various reproductive events, including sperm motility and capacitation, cell protection, acrosome reaction, fertilization and embryonic development. Empirical associations between seminal proteins and metabolites and fertility indicate that these molecules are potential molecular markers of male reproductive status in cattle and other species.

## Introduction

Pregnancy after artificial insemination (AI) is the best indicator of reproductive potential of sires. However, this information usually becomes available only after bulls are mature and have been selected for commercial use in the AI industry. Moreover, criteria such as sperm motility and morphology have limited associations with sire fertility, particularly in bulls selected by the AI industry (Killian *et al*., 1993; Moura, 2005; Moura *et al*., 2006; Oliveira *et al*., 2013; Dogan *et al*., 2015; Kaya and Memili, 2016). There can be substantial differences in fertility among bulls with normal semen parameters and those with non- compensable sperm defects may never achieve adequate fecundity (Oliveira *et al*., 2013; Dogan *et al*., 2015; Kaya and Memili, 2016). Therefore, mechanisms by which sperm molecular profiles influence bull fertility are not fully understood. In this context, there are efforts to identify molecular markers of gamete function in farm animals and humans. Candidate makers include sperm RNA, proteins and various molecules in reproductive fluids. These studies are based on the hypothesis that molecular components of sperm and/or from the surrounding media influence fertilizing capacity. In this regard, analysis of seminal plasma proteome and metabolome will provide information about mechanisms regulating sperm fertilizing potential and reproductive performance. Thus, the present review discusses the roles of selected seminal plasma proteins and metabolites and how their expression relates to fertility, especially in cattle.

## Seminal plasma proteins

### Proteins involved in sperm protection

Seminal plasma contains proteins that protect sperm in the epididymis (Hinton *et al*., 1995; Kraus *et al*., 2005), after ejaculation and in the female reproductive tract. Production of reactive oxygen species (ROS) is a component of sperm physiology (MacLeod, 1943); however, excessive ROS disturbs sperm homeostasis through formation of lipid peroxidation, reduction of enzymes that regulate calcium influx, and loss of ATP (Ohta *et al*., 1989; Aitken *et al*., 1993). To mitigate deleterious effects of excessive ROS, the epididymis secretes antioxidant enzymes (Hinton *et al*., 1996), including glutathione S- transferase, tioredoxin peroxidase, superoxide dismutase, glutathione peroxidase (GSHPx) and catalase (Alvarez and Storey, 1983; Jeulin *et al*., 1989; Fouchécourt *et al*., 2000; Dacheux *et al*., 2006). Of these, GSHPx catalyzes the reduction of hydrogen peroxide (Halliwell and Gutteridge, 1990), protecting sperm against excessive ROS (Perry *et al*., 1992; Dacheux *et al*., 2005). For example, increased GSPHx activity in ram semen is linked to maintenance of sperm viability (Casao *et al*., 2010). Another seminal plasma molecule, acidic seminal fluid protein (aSFP), also controls oxidative stress in the bovine reproductive tract (Einspanier *et al*., 1993; Schöneck *et al*., 1996). This protein shares identity with molecules of the spermadhesin family (Romão *et al*., 1997) and, in the bull, is secreted by the epididymis and accessory sex glands (Moura *et al*., 2007a, 2010). Although aSFP binds to ejaculated sperm, it is lost after capacitation (Dostàlovà *et al*., 1994). Therefore, unlike porcine spermadhesins (Caballero *et al*., 2004, 2005), it appears that bovine aSFP does not participate in sperm-oocyte interactions. However, aSFP has been associated with survival of cryopreserved bull sperm (Jobim *et al*., 2004).

Ion chelators in seminal plasma, such as lactoferrin (LF), also protect sperm against effects of lipid peroxidation (Ochsendorf, 1999). Lactoferrin sequesters ionic iron (Nozaki *et al*., 2003) and adsorption to sperm during epididymal transit (Jin *et al*., 1997) and ejaculation (Thaler *et al*., 1990). In stallions, LF represents 41.2% of all proteins secreted by the epididymis (Fouchecourt *et al*., 2000) and high concentrations of LF in horse and dog seminal plasma relate to total number of sperm (Kikuchi *et al*., 2003a, b). Seminal albumin, in turn, binds to lipid peroxides, contributing to sperm protection (Alvarez and Storey, 1983) and is positively correlated with percentage of morphologically normal sperm in bovine semen (Elzanaty *et al*., 2007).

Clusterin, another seminal plasma molecule with protective roles, acts as a chaperone (Humphreys *et al*., 1999) and inhibits cell lysis by complement- mediated mechanisms present in female secretions (Ibrahim *et al*., 1999; Meri and Jarva, 2001). In the epididymis, clusterin affects maturation, lipid transport (Tenniswood *et al*., 1992) and sperm membrane remodeling (Humphreys *et al*., 1999). Clusterin chaperone activity is consistent with its ability to interact with various types of proteins *in vivo* (Carver *et al*.; 2003). *In silico* analysis of clusterin networking indicates potential interactions with proteases and protease inhibitors, such as plasminogen, alpha-2- macroglobulin, TIMP-1, alpha-2-antiplasmin precursor and plasminogen activator inhibitor 1. Clusterin also has putative links to fibronectins, which participate in cell adhesion, wound healing and maintenance of cell structure, including GTP protein-coupled receptors and modulators of cell growth. Seminal plasma clusterin is inversely related to percentage of sperm with intact membrane in peccaries (*Peccari tajacu* L.; Santos *et al*., 2014) and with percentage of morphologically normal sperm in semen from Brahman bulls (Boe- Hansen *et al*., 2015). In contrast, bull and ram sperm with morphologic defects have extensive clusterin binding (Ibrahim *et al*., 2001a, b). This association probably occurs as a result of clusterin´s ability to bind to damaged portions of hydrophobic regions of sperm membranes (Bailey and Griswold, 1999). Sertoli cell- secreted clusterin prevented apoptosis in rat testes subjected to hyperthermia (Matsushita *et al*., 2016) and in humans, clusterin secreted in the fluid of the seminiferous epithelium has positive associations with fertility (Salehi *et al*., 2013). In addition, seminal clusterin promotes immune tolerance to male antigens in humans, mitigating female immune reactions to male factors (Merlotti *et al*., 2015). High levels of clusterin are associated with advanced physiopathological states, such as kidney diseases, neurodegenerative disorders, artheriosclerosis, heart attack and cancer (Wehrli *et al*., 2001; Trougakos *et al*., 2002; Pucci *et al*., 2004; Calero *et al*., 2005). We have characterized the seminal plasma proteome of several domestic and wild species, including bulls (Moura *et al*., 2007a; Rego *et al*., 2014, 2016; Menezes *et al*., 2017), rams (Souza *et al*., 2012), boars (González-Cadavid *et al*., 2014), peccaries (Santos *et al*., 2014), dogs (Aquino-Cortez *et al*., 2017) and coatis (Silva *et al*., 2018), among others. Clusterin is present in the semen of all these species, in moderate to high concentrations. Thus, many animals have a clusterin-based, conserved mechanism for sperm protection and regulation of immune reactions initiated by male gametes in the female reproductive tract.

### Proteins associated with sperm motility

Seminal plasma contains various proteins associated with sperm motility (Baas *et al*., 1983), e.g., kallikrein-cinins. In this case, kininogenin seminal plasma is a specific substrate for kalikrein (Fink *et al*., 1989), an important stimulator of post-ejaculation sperm motility (Schill *et al*., 1989). There is a positive correlation between seminal plasma kallikrein activity and sperm motility, with exogenous kallikrein enhancing bovine sperm motility (Somlev *et al*., 1996). Angiontensin converting enzyme (ACE) is another seminal plasma component related to the kalikrein system (Hohlbrugger*et al*., 1984). This enzyme catalyzes formation of angiontensin II and binds to receptors on sperm, enhancing motility (Vinson *et al*., 1996). Furthermore, ACE activity in ram seminal plasma is positively correlated with sperm concentration and fertility (Métayer *et al*., 2001; Gatti *et al*., 2004). In contrast, inhibition of ACE activity in bovine seminal plasma decreases progressive motility and inhibits acrosome reaction after *in vitro* capacitation (Costa and Thundathil, 2012).

### Proteins involved in sperm capacitation, acrosome reaction and fertilization

Phospholipid binding proteins belonging to the family of BSPs (Binder of Sperm Proteins) are present in seminal plasma of several species, including bulls, bucks (male goats and rabbits), rams, rodents, stallions and men (Moura *et al*., 2007a; Manjunath *et al*., 2009; Souza *et al*., 2012; Plante *et al*., 2016). BSPs comprise ~60% of all proteins of the accessory sex gland fluid (Moura *et al*., 2007a) and seminal plasma (Manjunath and Sairam, 1987) of *Bos taurus* bulls and nearly the same amount in seminal plasma of *Bos indicus* bulls (Rego *et al*., 2014). In cattle, BSP proteins are secreted as isoforms with 14-15 kDa (BSP1 and BSP3) and 30 kDa (BSP5). Both BSP1 and BSP5 have two fibronectin type II domains arranged in tandem and amino terminal extensions that are O-glycosylated at threonine residues. Such biochemical attributes allow BSP1 and BSP5 to interact with sperm and to modulate ligand-binding activities by similar mechanisms (Calvete *et al*., 1996; Manjunath *et al*., 2009), with functional similarities (Manjunath and Thérien, 2002). Bovine BSPs are typical accessory sex gland proteins (Manjunath and Thérien, 2002; Moura *et al*., 2007a, 2010). BSPs bind to bull sperm at ejaculation (Manjunath and Thérien, 2002) and remain there after sperm contact oviductal secretions *in vitro* (Souza *et al*., 2008), as well as in acrosome-reacted or frozen-thawed sperm (Rodriguez- Villamil *et al*., 2016). BSPs also mediate the interaction between sperm and the oviduct epithelium (Gwathmey *et al*., 2006; Suarez, 2016). The most studied role of BSPs is their ability to bind and remove phospholipids and cholesterol from the sperm membrane, an initial event of capacitation (Thérien *et al*., 1999). Capacitating effects of BSPs have been reported in other species, including mice (Plante and Manjunath, 2015) and humans (Plante *et al*., 2014). However, while ruminant BSPs originate mainly from the accessory sex glands (Manjunath *et al*., 2009; Souza *et al*., 2012; van Tilburg *et al*., 2014), they are expressed in the epididymis of mice and humans.

In addition to sperm capacitation, BSP1 affects *in vitro* fertilization and embryonic development. The study conducted by Rodriguez-Villamil *et al*. (2016) evaluated cumulus-oocyte complexes (COCs) incubated with frozen-thawed ejaculated sperm (18 h) in Fert- TALP medium containing: heparin and BSP1. With ejaculated sperm, cleavage rates were similar when Fert-TALP medium was incubated with heparin, 10 or 20 µg/ml BSP1. Day-7 blastocyst rates were equivalent after incubations with heparin or 10 µg/ml BSP1, but there were marked reductions in blastocyst formation after IVF media were supplemented with 20 or 40 µg/ml. Therefore, BSP1 is as efficient as heparin for inducing capacitation and fertilizing capacity of frozen- thawed ejaculated sperm. However, damage caused to embryo development may have been caused by BSP1 itself. High concentrations of and/or prolonged exposure to BSP proteins are harmful to cryopreserved sper due to membrane destabilization and excessive phospholipid and cholesterol efflux (Thérien *et al*., 1995, 1998; Manjunath and Thérien, 2002). Furthermore, content of BSP5 in accessory sex gland fluid has a quadratic association with bull fertility (Moura *et al*., 2006), suggesting that excessive BSP was detrimental to sperm physiology and/or embryo development.

In the same study (Rodriguez-Villamil *et al*., 2016), cleavage rates were higher after IVF using cauda epididymal sperm and any BSP1 concentration (10, 20 or 40 µg/ml) embryo development (day 8) was greater after inclusion of 20 or 40 µg/ml BSP1 in the IVF media, with or without heparin. Thus, we concluded that: 1) heparin has limited effect on cauda epididymal sperm *in vitro* (based on fertilization rates and blastocyst formation; 2) BSP1 has better effects on embryo growth than heparin; and 3) a combination of BSP1 with heparin does not enhance cleavage rates and embryo development beyond those obtained with BSP1. We also verified that SP1 did not cause reductions in bovine blastocyst growth after IVF with epididymal sperm, in contrast to results obtained with ejaculated sperm. Therefore, previous exposure of sperm or not to seminal plasma determines how sperm will respond to BSP in vitro. Additionally, combining heparin and BSP1 did not increase capacitation rates of ejaculated sperm. And both cleavage rates and blastocyst growth were similar after ejaculated sperm were incubated with heparin, BSP-1+heparin or BSP-1. With epididymal sperm, the best results or capacitation and blastocyst growth were obtained with BSP-1, when compared to heparin and heparin+BSP-1 (Rodriguez-Villamil *et al*., 2018; Federal University of Ceara, Fortaleza, Brazil; unpublished data). Thus, BSP-1 is a potent capacitating factor for bovine sperm and it increases fertilization rates, with no dependence on heparin.

Despite multiple beneficial roles of BSPs, these molecules can damage sperm during cryostorage, as they extract phospholipids and cholesterol from the membrane in a concentration- and time-dependent manner (Manjunath *et al*., 2002; Plante *et al*., 2016). Such deleterious effects occur when sperm are exposed for prolonged periods and/or to excessive concentrations of BSPs. In this regard, extenders used for sperm preservation, such as egg-yolk (EY) and milk, contain components that associate with BSPs (Manjunath *et al*., 2002). There are interactions between low-density lipoproteins in EY (Bergeron and Manjunath, 2006) or milk proteins; the latter can prevent excessive BSP binding to sperm and excessive phospholipid removal from the membrane, thereby protecting sperm during cryopreservation (Plante *et al*., 2015). In goats, milk proteins (casein and β-lactoglobulin) bind to BSPs and reduces BSP interactions with sperm (Menezes *et al*., 2016). Currently, BSPs are one of the most studied mammalian seminal plasma proteins and effects on ejaculated sperm, including capacitation, interaction with the oviduct epithelium and fertilization. That BSPs interact with components of semen extenders suggest that these proteins are potential targets for development of biomolecules that could enhance assisted reproductive technologies.

Seminal plasma phospholipase A2 (PLA2) participates in capacitation, acrosome reaction and sperm-oocyte membrane fusion (Soubeyrand *et al*., 1997; Pietrobon *et al*., 2005; Roldan and Shi, 2007), promotes release of fatty acids and phospholipids involved in final stages of gamete fusion (Roldan, 1998) and has antimicrobial effects. Furthermore, its expression in bovine seminal plasma is associated with fertility (Moura *et al*., 2006). Osteopontin (OPN) concentrations in bovine seminal plasma were related to *in vivo* fertility of Holstein bulls (Killian *et al*., 1993; Moura *et al*., 2006) and to fertilizing capacity of cauda epididymal sperm treated with accessory sex gland fluid in IVF trials (Henault *et al*., 1995; Moura *et al*., 2007b). OPN is mainly secreted by the accessory sex glands and binds to sperm after ejaculation and after they contact secretions of the oviduct and are capacitated (Souza *et al*., 2008). Also, OPN has a calcium binding site and a domain to link with heparin, consistent with its effects on sperm capacitation (Monaco *et al*., 2009; Boccia *et al*., 2013).

Alterations in the OPN gene reduce seminal plasma OPN concentration (Rorie *et al*, 2016). Furthermore, in IVF studies, percentage of fertilized bovine oocytes was significantly reduced by addition of OPN antibodies to fertilization media and exposure of sperm or oocytes to antibodies against alpha V and alpha5 integrins before fertilization (Gonçalves *et al*., 2007). Also, pre-treatment of bovine sperm and oocytes with OPN enhances both *in vitro* fertilization and early embryo development (Gonçalves *et al*., 2008a, b). The RGD amino acid sequence of osteopontin mediates its link with α5 and αv integrins (Denhardt, 2002; Wai and Kuo, 2004) and the ability of osteopontin to support cell adhesion is prevented when the RGD sequence is mutated (Liaw *et al*., 1995; Xuan *et al*., 1995). Treatment of sperm or oocytes with an RGD peptide, but not with an RGE sequence, reduced both the number of sperm bound to the zona pellucida and fertilization rates, similar to effects of anti-osteopontin antibodies. It appears that OPN interacts with sperm through integrins (Gonçalves *et al*., 2007). Incubation of oocytes with osteopontin purified from bovine milk increased cleavage rates on day 4, blastocyst development on day 8 and hatched blastocysts on day 11 (Gonçalves *et al*., 2007). Furthermore, OPN purified from milk improved sperm capacitation and addition of OPN to IVF media enhanced bovine blastocyst formation (Monaco *et al*., 2009). Moreover, in an IVF system, using frozen- thawed bull semen, OPN improved fertilization rates and blastocyst development on day 8 (Gonçalves *et al*., 2008a). In swine, supplementation of fertilization media with recombinant rat OPN enhanced fertilization rates by 41% and reduced polyspermy (Hao *et al*., 2006). Exogenous OPN added to IVF media improved cleavage rates and swine embryo development, and inhibited apoptosis and DNA fragmentation (Hao *et al*., 2008). Moreover, anti-OPN antibodies decreased rates of *in vitro* fertilization and blastocyst growth in mice (Liu *et al*., 2015). Clearly, OPN affects fertilization and post-fertilization events.

Osteopontin is typically involved in cell adhesion, tissue and extracellular remodeling, inflammation and immune-mediated events (Denhardt, 2002; Wai and Kuo, 2004; Rittling and Singh, 2015; Bouleftour *et al*., 2016). Despite substantial knowledge regarding actions of osteopontin in several tissues, an understanding of its function in male reproduction is far from complete. There is general consensus that OPN secreted by the accessory sex glands binds to sperm during ejaculation through integrins and that the integrin-OPN complex interacts with the zona pellucida (D’Cruz, 1996). This model is supported by the presence of OPN in bovine oviductal fluid (Gabler *et al*., 2003). Additionally, OPN binds to the CD44 receptor, which usually participates in cell adhesion (Cichy and Puré, 2003), and has been expressed in sperm (Bains *et al*., 2002) and oocyte membranes (Schoenfelder and Einspanier, 2003). In the bull, OPN binds to the acrosome at ejaculation (Cancel *et al*., 1999) and this sperm-OPN link is preserved after sperm contacts with oviductal fluid and undergoes an acrosome reaction *in vitro* (Souza *et al*., 2008). In addition to sperm binding, OPN interacts with the zona pellucida and oolemma of bovine oocytes (Souza *et al*., 2008). Consequently, we propose that OPN adheres to sperm and this complex connects to the zona pellucida or to OPN-zona pellucida, as OPN can form high-affinity bonds with other OPN molecules (Kaartinen *et al*., 1999;

Goldsmith *et al*., 2002). When entering the periviteline space, OPN attached to the post-equatorial segment would mediate the interaction of sperm and oolema, also through integrins and/or CD44. Integrins (αv and α5) are present in bovine (Erikson *et al*., 2008) and human sperm (Fusi *et al*., 1996; Reddy *et al*., 2003), as well as on human oolema (D’Cruz, 1996) and CD44 transmembrane glycoproteins are present in bovine sperm and oocytes. Interactions of sperm OPN with oocyte integrins and CD44 receptors could trigger intracellular signaling, as reported for other cell types (Wai and Kuo, 2004; Rangaswami *et al*., 2006), and affect fertilization and early embryo development.

## Metabolomics

Metabolites are the result of metabolic reactions associated with various biochemical pathways (Dunn *et al*. 2011). Many of these molecules have important roles in biological processes and represent potential biomarkers for predicting or detecting developmental states, physiological events, diseases or specific phenotypes. Therefore, metabolomics is used to understand networks of metabolites and have provided comprehensive identification and quantification of small molecules, including amino acids, peptides, vitamins, minerals, lipids, and carbohydrates in diverse cells, tissues, fluids, organs and organisms (Oliver *et al*., 1998; Fiehn 2001, 2002; Dunn *et al*., 2005).

Substantial progress has been made in the study and development of methodological strategies for using metabolomics, metabonomics, metabolic- fingerprinting, metabolite targeting, and metabolic profiling. Metabonomics is used to measure differences in levels of metabolites resulting from such factors as pathological or genetic changes, toxins, or use of drugs. Metabolic fingerprinting is a rapid method to evaluate and classify biologic samples or biopsies. Furthermore, metabolite target analysis is used to identify specific metabolic pathways of a limited number of metabolites, whereas metabolic profiling evaluates a cluster of metabolites that participate in a specific metabolic pathway (Dunn and Ellis, 2005; Hollywood *et al*., 2006; Holmes *et al*., 2008; Dunn *et al*., 2011; Patti *et al*., 2010). Metabolomics can be performed associated with other omics approaches, e.g. genomics, transcriptomics, and proteomics. For example, an Integrative Personal Omics Profile (iPOP) to identify markers for possible diseases affecting an individual could lead to an early diagnosis and perhaps prevention of certain diseases (Chen *et al*., 2012). Although complementary to other omics, metabolomics provides identification and quantification of products from metabolism and its pathways, analyses of modifications in metabolic reactions, and characterization of phenotypes and identification of potential biomarkers for such phenotypes (Fiehn, 2001, 2002; Dunn *et al*., 2005; Goodacre *et al*., 2004; Hollywood *et al*., 2006; Patti *et al*., 2010).

### Metabolomics and reproductive biology

Advanced and more sensitive methods are vital for addressing major questions in biology and biotechnology, including those related to assisted reproductive technologies (ART). It is well known that metabolites have critical roles in specific pathways related to fertilization, implantation and embryonic development. Some techniques used in metabolome analysis for studies of reproductive biology include proton nuclear magnetic resonance (1H NMR), mass spectrometry (MS), fourier transform infrared spectroscopy (FTIR), near infrared (NIR) and Raman (Singh and Sinclair, 2007, Seli *et al*., 2010a; Kovac *et al*., 2013; Muñoz *et al*., 2014a; b). Metabolomics methods have been used as noninvasive approaches to improve assessment of embryo quality (Singh and Sinclair, 2007; Bromer and Seli, 2008; Nagy *et al*., 2008; Seli *et al*., 2010a; Montag *et al*., 2013). For example,1H NMR scans compared metabolomes in the culture media for human embryos before transfer. In that study, glutamate was associated with subsequent developmental potential (Seli *et al*., 2008). In addition, NIR, Raman and 1H NMR used for metabolome analysis of human embryo culture media were valuable for predicting successful implantation and pregnancy after IVF (Seli *et al*., 2007, 2008, 2010b). FTIR metabolomics were used to determine gender of bovine embryos (Muñoz *et al*., 2014a, b). This is also an effective and non-invasive method to determine embryo viability and the metabolic profile of blood plasma from recipient cows. Further, FTIR can be used to identify superior embryos and recipient females for optimum pregnancy outcome (Muñoz *et al*., 2014b).

As indicated above, methods of conventional semen evaluation most often give only descriptive information and have limitations to predict in fertility. However, various molecular approaches, such as metabolomics, have provided more in depth understanding of mechanisms causing male infertility (Deepinder *et al*. 2007; Aitken, 2010). Metabolomics has promise in identifying potential biomarkers of male fertility and infertility (Gilany *et al*., 2014; Goodacre *et al*., 2004; Deepinder *et al*., 2007; Kovac *et al*., 2013). The presence or changes in specific metabolites could be related to male gamete functions, perhaps enabling evidence-based techniques to prevent or mitigate infertility (Aitken, 2010). Metabolomics approach using Raman spectroscopy to analyze human seminal plasma facilitated diagnosis of normospermic and asthenozoospermic men (Gilany *et al*., 2014). Furthermore, 1H NMR identified fertility-associated biomarkers in seminal plasma and serum of high- and low-fertility bulls. Metabolites, such as citrate, tryptamine, taurine, and leucine were identified in seminal plasma, whereas asparagine, glycogen, citrulline, and isoleucine were present in serum (Kumar *et al*., 2015). Using 1HNMR, Hamamah *et al*. (1998) detected increased choline/citrate, choline/lactate, and glycerophosphorylcholine/choline ratios in seminal plasma of men afflicted with spermatogenic failure versus those with obstructive azoospermia. Several small molecular markers were identified in the urine of men with normozoospermic infertility using liquid chromatography-mass spectrometry (LC-MS) in combination with bioinformatics and multivariate analyses. In this research, leukotriene E4,3- hydroxypalmitoylcarnitine, aspartate, xanthosine, and methoxytryptophan were biomarkers of infertility (Zhang *et al*., 2014). Clearly, metabolomics can be used to identify molecular markers of male fertility.

## Conclusions

In recent decades, methods in proteomics and metabolomics have enabled detection of unprecedented numbers of molecules in the seminal plasma of farm animals, wild species and humans. This broadens our knowledge regarding roles of these molecules and their contributions to male fertility. Metabolomics can identify numerous classes of substances associated with metabolic pathways, leading to challenges in interpretation. Empirical associations exist between specific seminal proteins (Table 1), metabolites and fertility indexes. Experiments also confirm cause and effect relations between seminal plasma proteins (e.g.osteopontin and BSPs) and IVF and early embryo development, suggesting that seminal proteins have potential in animal biotechnology.

Studies to describe components of the seminal plasma are vital to construct comprehensive libraries of seminal plasma compounds. As many as 4,000 proteins have already been identified in human seminal plasma, although there may be up to 10,000 present (Gilany *et al*., 2014). A human proteome atlas (https://www.proteinatlas.org/) of human tissues and organs is under development (Omenn *et al*., 2017; Uhlén *et al*., 2015; Thul *et al*., 2017). Fundamental research sets the foundation of science and technology. However, investigations that use omics approaches and reproduction need to be focused on finding markers of traits that are important for livestock industry in different regions of the world. Lessons could be learned from translational research, where research is carried out by multidisciplinary teams, joining efforts from basic science, applied investigators and professionals in the front of technology.

**Table 1:** Functional groups and mechanism of actions of major seminal plasma proteins.

Major functional group	Protein	Mechanism of action	Major references
Proteins involved in sperm protection	Glutathione peroxidase Acidic seminal fluid protein Lactoferrin	Catalyzes reduction of H_2_O_2_, protects sperm against excessive ROS. Sperm decapacitation, oxidative stress control, survival of cryopreserved sperm Ion chelators, protects sperm against effects of lipid peroxidation	(Halliwell and Gutteridge, 1990; rams); (Perry *et al*., 1992; Dacheux *et al*., 2005; bulls) (Einspanier *et al*., 1993; Schöneck *et al*., 1996; bull); (Dostàlovà *et al*., 1994; bull); (Jobim et al., 2004; bull) (Ochsendorf, 1999; men)
	Albumin	Binds to lipid peroxides in sperm membrane, sperm protection	(Alvarez and Storey, 1983; rabbit)
	Clusterin	Chaperone and sperm membrane remodeling, protects against female reproductive tract immune response, binds to damage sperm membrane	(Humphreys *et al*., 1999; men); (Ibrahim *et al*., 1999; bull; Merlotti *et al*., 2015; men); (Bailey and Griswold, 1999; rat)
Proteins associated with sperm motility	Kallikrein-cinins Angiotensin converting enzyme	Substrate for kallikrein, which enhance sperm motility Catalyzes angiotensin II formation, Enhance motility.	(Somlev *et al*., 1996; bull) (Vinson *et al*., 1996; rat and men; Costa and Thundathil, 2012; bull)
Proteins involved in sperm capacitation, acrosome reaction and fertilization	Binder of sperm proteins Phospholipase A2 Osteopontin	Bind to sperm at ejaculation; mediate gametes interaction, phospholipids and cholesterol efflux from sperm membrane; enhance in vitro fertilization and embryonic development. Release of fatty acids and phospholipids involved in final stages of gamete fusion; antimicrobial effects. Binds to sperm acrosome after ejaculation; sperm capacitation; interacts with sperm through integrins, interacts with zona pellucida and oolemma; binds to sperm CD44 receptor; enhances *in vitro* fertilization and embryo development; reduces polyspermy and inhibits apoptosis and DNA fragmentation.	(Manjunath and Thérien, 2002; bull); (Gwathmey *et al*., 2006; bull); (Thérien *et al*., 1999; bull) (Rodriguez-Villamil *et al*., 2016; bull); Manjunath *et al*., 2002; bull; Plante *et al*., 2016; bull) (Soubeyrand *et al*., 1997; bull; Pietrobon *et al*., 2005; mouse; Roldan and Shi, 2007); Moura *et al*., 2006; bull) (Souza *et al*., 2008; bull; Cancel *et al*., 1999; bull); (Boccia *et al*., 2013; buffalo; Monaco *et al*., 2009; bull); (Gonçalves *et al*., 2007; bull; D’Cruz, 1996; men); (Souza *et al*., 2008; bull); (Cichy and Puré, 2003; men); (Gonçalves *et al*., 2008a; bull); (Hao *et al*., 2006; swine; Hao *et al*., 2008; swine)
